# A Manual of Chemistry, Inorganic and Organic

**Published:** 1905-12

**Authors:** 


A MANUAL OF CHEMISTRY, INORGANIC AND ORGANIC. By Authur P. Luff, M.D., B.Sc. (Lond.) F.R.C.P., F.I.C., and Frederic James M. Page, B.Sc. (Lond.), F.I.C. Illustrated with 43 engravings. Third edition. Revised throughout. W. T. Keener & Co. Chicago. Price $1.75 net.

This little though comprehensive work has been rearranged and brought up to date. It is intended as a guide for the use of the medical student in the study of chemistry, and undoubtedly fulfills this purpose. It brings together in a concise form those portions of chemical science that directly or indirectly bear on the study of medicine.

---

### Preliminary Announcement.

Messrs. D. Appleton & Co. announce that they expect to publish at an early date a new book entitled "Differential Diagnosis and Treatment of Disease." The author is Augustus Caillé, Fellow of the New York Academy of Medicine; Member and ex-President of the American Pediatric Society; Professor of Diseases of Children, New York Post-Graduate Medical School and Hospital; Visiting Physician to the New York Post-Graduate and German Hospitals.

